# Nutrient Availability Does Not Affect Community Assembly in Root-Associated Fungi but Determines Fungal Effects on Plant Growth

**DOI:** 10.1128/msystems.00304-22

**Published:** 2022-06-13

**Authors:** Jose G. Maciá-Vicente, Bing Bai, Run Qi, Sebastian Ploch, Florian Breider, Marco Thines

**Affiliations:** a Institute of Ecology, Evolution and Diversity, Goethe University Frankfurt, Frankfurt am Main, Germany; b Plant Ecology and Nature Conservation, Wageningen University & Research, Wageningen, The Netherlands; c Department of Microbial Ecology, Netherlands Institute for Ecology (NIOO-KNAW), Wageningen, The Netherlands; d Wageningen Seed Laboratory, Laboratory of Plant Physiology, Wageningen University & Research, Wageningen, The Netherlands; e University of Copenhagen, Department of Biology, Copenhagen N, Denmark; f Plant-Microbe Interactions, Department of Biology, Faculty of Science, Utrecht University, Utrecht, Netherlands; g Biodiversity and Climate Research Centre (BiK-F), Senckenberg Gesellschaft für Naturforschung, Frankfurt am Main, Germany; h LOEWE Centre for Translational Biodiversity Genomics, Frankfurt am Main, Germany; i School of Architecture, Civil and Environmental Engineering (ENAC), Ecole Polytechnique Fédérale de Lausanne, Lausanne, Switzerland; Oak Ridge National Laboratory

**Keywords:** *Arabidopsis thaliana*, fungi, grasslands, growth promotion, heathlands, nutrient uptake, roots

## Abstract

Nonmycorrhizal root-colonizing fungi are key determinants of plant growth, driving processes ranging from pathogenesis to stress alleviation. Evidence suggests that they might also facilitate host access to soil nutrients in a mycorrhiza-like manner, but the extent of their direct contribution to plant nutrition is unknown. To study how widespread such capacity is across root-colonizing fungi, we surveyed soils in nutrient-limiting habitats using plant baits to look for fungal community changes in response to nutrient conditions. We established a fungal culture collection and used Arabidopsis thaliana inoculation bioassays to assess the ability of fungi to facilitate host’s growth in the presence of organic nutrients unavailable to plants. Plant baits captured a representation of fungal communities extant in natural habitats and showed that nutrient limitation has little influence on community assembly. Arabidopsis thaliana inoculated with 31 phylogenetically diverse fungi exhibited a consistent fungus-driven growth promotion when supplied with organic nutrients compared to untreated plants. However, direct phosphorus measurement and RNA-seq data did not support enhanced nutrient uptake but rather that growth effects may result from changes in the plant’s immune response to colonization. The widespread and consistent host responses to fungal colonization suggest that distinct, locally adapted nonmycorrhizal fungi affect plant performance across habitats.

**IMPORTANCE** Recent studies have shown that root-associated fungi that do not engage in classical mycorrhizal associations can facilitate the hosts’ access to nutrients in a mycorrhiza-like manner. However, the generality of this capacity remains to be tested. Root-associated fungi are frequently deemed major determinants of plant diversity and performance, but in the vast majority of cases their ecological roles in nature remain unknown. Assessing how these plant symbionts affect plant productivity, diversity, and fitness is important to understanding how plant communities function. Recent years have seen important advances in the understanding of the main drivers of the diversity and structure of plant microbiomes, but a major challenge is still linking community properties with function. This study contributes to the understanding of the cryptic function of root-associated fungi by testing their ability to participate in a specific process: nutrient acquisition by plants.

## INTRODUCTION

Fungi are key drivers of soil nutrient cycles, acting as major decomposing agents of plant biomass, storing massive amounts of nutrients in their mycelium and helping relocate those nutrients between the mineral and organic fractions of soil (1). Through their associations with roots, soil fungi can contribute most of the nutrients taken up by plants, as well as act as mediators in the belowground trade of photoassimilates between neighboring trees (2, 3). The main fungal players in these processes are those that establish mycorrhizal associations with roots, involving the formation of specialized interfaces for the active exchange of resources with the host (4). Such a lifestyle has independently evolved multiple times across the fungal tree of life (5), showing that it is a rewarding habit and that fungi possess traits that favor their engagement in nutritional symbioses with plants. Nevertheless, most members of fungal communities in soil and roots are not mycorrhizal or have unknown mycorrhizal status (6). They are often implicated in processes that influence plant growth, from pathogenesis to stress alleviation (7, 8), but knowledge of their direct contribution to plant nutrition is, at best, fragmentary.

Nonmycorrhizal, root-associated fungi have long been hypothesized to participate in the acquisition of nutrients by their plant hosts, mainly via their saprotrophic break-down of soil organic matter and the subsequent release of nitrogen (N) and phosphorus (P) sequestered therein (9–11). Indirect evidence for this assumption was provided by studies involving soil amendments with organic nutrient sources, in which fungus-inoculated plants showed growth enhancement compared to uninoculated plants (12). More recently, several tracer isotope studies have demonstrated that phylogenetically diverse fungi can actively translocate N or P from the substratum to plant roots. For example, Behie et al. (13) showed that the soilborne entomopathogenic fungus Metarhizium robertsii can transfer N from insect carcasses to several plant species. Hill et al. (14) demonstrated an analogous N transfer from soil organic matter by a *Tapesia* sp. root endophyte. Similarly, Hiruma et al. (15) and Almario et al. (16) found that unrelated endophytes of nonmycorrhizal *Brassicaceae* can supply inorganic phosphate to plant hosts subject to P starvation. Such direct transfer of nutrients is reminiscent of intermediate evolutionary stages toward mycorrhizal lifestyles, but genome sequence data instead suggest that root endophytes may be on different adaptive paths, since they retain and even have expanded genomic toolboxes for plant decay that tend to be lost in biotrophic mycorrhizal fungi (16–20). While the evidence that some nonmycorrhizal fungi can actively contribute to their hosts’ nutrition is compelling, whether this ability is widespread across root-colonizing fungi and whether it importantly determines plant performance in nature remain unknown.

We aimed to identify groups of root-associated fungi potentially implicated in plant responses to nutrient limitation and to evaluate how common the ability to assist plant hosts in the uptake of nutrients is among fungi. First, we studied the impact of contrasting nutrient availability conditions on root-associated fungal community assembly, and we isolated candidate strains using a plant bait bioassay inspired by those used to capture arbuscular mycorrhizal fungi (21). Because plants can actively regulate root colonization by fungi (including nonmycorrhizal fungi) (15, 22, 23), we hypothesized that starved roots would become enriched in particular fungal lineages involved in enhancing nutrients uptake, thereby enhancing our chances to isolate fungi with relevant roles in plant nutrition. We looked for consistent patterns of variation in fungal communities’ structure and composition across soils collected from seminatural heathlands and grasslands along a latitudinal gradient that share factors imposing nutrient limitations (24) and host locally specific fungal communities (25), while being subject to broadly variable environmental, geographic, and historic conditions.

Second, we tested for the capacity of a phylogenetically diverse selection of fungal isolates to enhance growth of plants subject to low available nutrients but supplied with organic nutrient sources readily usable by fungi. We used inoculation bioassays of Arabidopsis thaliana with amendments of glutamic acid (GA) and phytic acid (PA), which respectively represent common sources of soluble, organic N and P in heathland and grassland soils but that are only taken up at low rates by most temperate plants (26–31). Our hypothesis was that the ability to assist hosts’ nutrition is not phylogenetically conserved, since currently known nonmycorrhizal fungi displaying such capacity belong in several different higher lineages (18). We relied on transcriptome analyses to identify signatures for nutrient deficiency alleviation in host plants in response to fungal colonization, as done elsewhere under similar experimental settings (15, 17, 32).

## RESULTS

### Baits for root-associated fungi.

**FIG 1:**
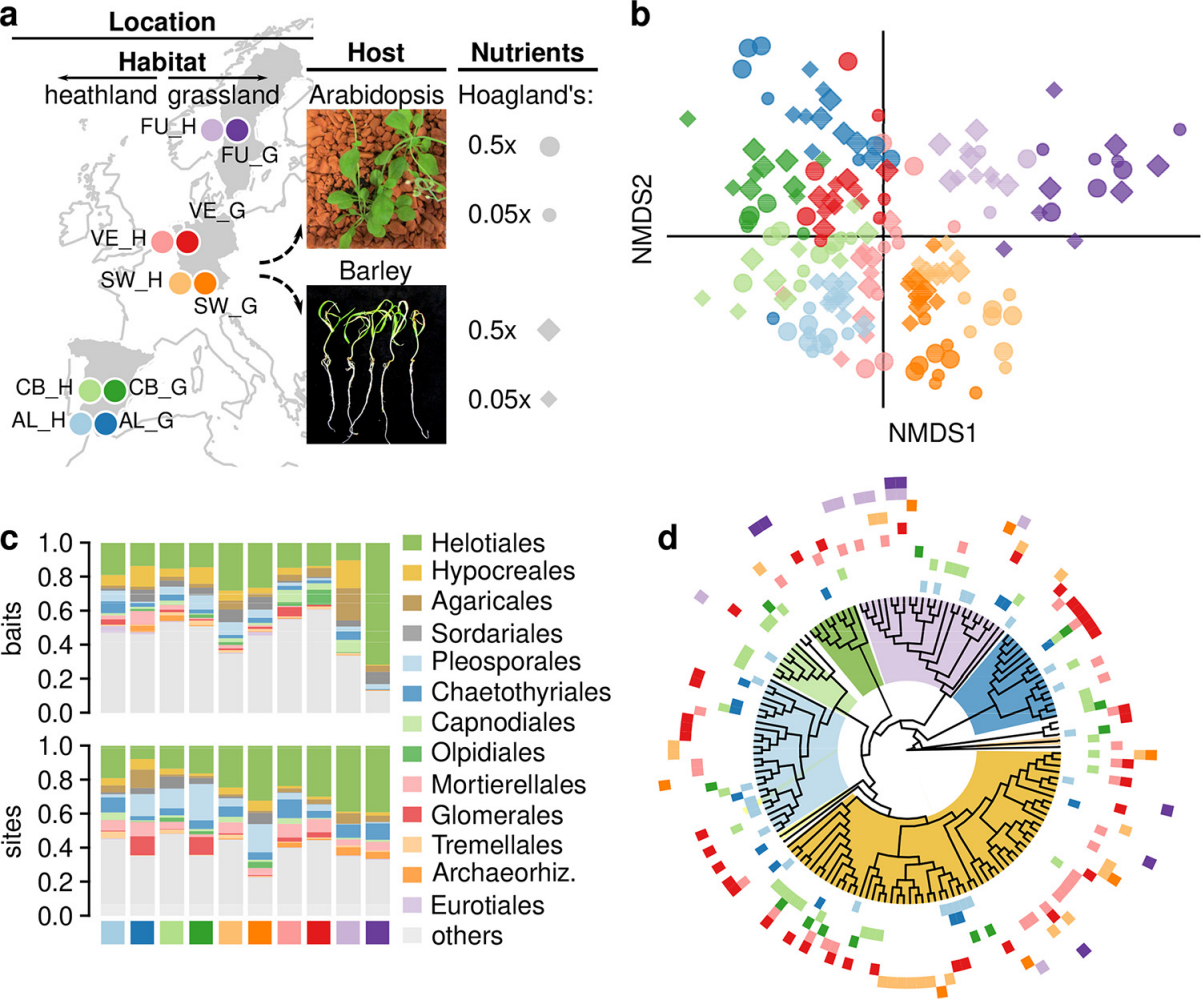
Assembly of fungal communities in root baits. (a) Experimental setup showing the geographical locations and habitat types of the sampling sites, and the plant host and nutrient conditions used in the root bait bioassays. See Table S1 at https://doi.org/10.6084/m9.figshare.14222264 for more details about the sampling sites. (b) Nonmetric multidimensional scaling (stress = 0.15) based on Bray-Curtis dissimilarities of fungal communities in root baits inoculated with nonsterilized soil. Point colors indicate location of origin, while shape and size indicate host plant and nutrient condition, as illustrated in panel a. (c) Proportion of read abundances at the order-level classification across sampling sites, found in root bait bioassays (top) and in soil and roots in the sampling sites of origin (bottom) (25). The 10 most frequent orders for either baits and original sites are shown, whereas the remaining orders are combined in “others.” (d) Phylogenetic tree based on the ITS and LSU rDNA regions of 152 OTUs representing all fungal cultures isolated from root baits. Clades encompassing OTUs in the main fungal orders are highlighted using the color key in panel c. The outer tracks indicate the sampling sites from where each OTU was isolated, with colors following the color key in panel a. See Fig. S4 for a detailed version of the tree.

We collected soil samples from seminatural heathlands and grasslands in five locations along a latitudinal gradient in Western Europe and used them to inoculate arabidopsis and barley roots in gnotobiotic bioassays subject to high or low nutrients (Fig. 1a). Both species showed decreased shoot biomass after 1 month under low nutrients compared to high-nutrient conditions (see Fig. S1 in the supplemental material), indicating an effective growth limitation by nutrients availability. These effects were independent of whether the soil inoculum was sterilized or not (see Fig. S1), but they were much more evident in arabidopsis [likelihood ratio test; *Χ^2^*(1) = 24, *P < *0.0001] than in barley [*Χ^2^*(1) = 9.4, *P = *0.002], indicating a stronger limitation in the former. The availability of nutrients did not significantly affect the growth of barley roots (see Fig. S1).

10.1128/msystems.00304-22.2 FIG S1 Growth response of bait plants Arabidopsis thaliana and *Hordeum vulgare* to inoculation with nonsterilized (NS) and sterilized (S) soil and different concentrations of nutrient solution. (a) Fresh weight of *A. thaliana* shoots. Values shown represent partial residuals, after accounting for the variation across sampling sites and experimental conditions. The box-and-whisker plots show the data distribution for each treatment, and points indicate the individual values. (b) Fresh weight of *H. vulgare* shoots. (c) Fresh weight of barley roots. Download FIG S1, PDF file, 0.06 MB. Copyright © 2022 Maciá-Vicente et al. 2022 Maciá-Vicente et al. https://creativecommons.org/licenses/by/4.0/ This content is distributed under the terms of the Creative Commons Attribution 4.0 International license.

10.1128/msystems.00304-22.5 FIG S4 Overlap between the fungal OTUs found in the root bait bioassays and those found in soil and roots in the sampling sites of origin. The values above bars indicate the percentage of OTUs in the root-baits that were also detected in the respective sites. Download FIG S4, PDF file, 0.02 MB. Copyright © 2022 Maciá-Vicente et al. 2022 Maciá-Vicente et al. https://creativecommons.org/licenses/by/4.0/ This content is distributed under the terms of the Creative Commons Attribution 4.0 International license.

We evaluated how factors related to sample origin, host plant, and nutrient availability affected the assembly of fungal communities in roots, using Illumina MiSeq amplicon sequencing. The data set included 2,199,173 high-quality reads and 1,572 fungal operational taxonomic units (OTUs), 1,103,464 and 99 of which were respectively found in control plants inoculated with sterilized soil and removed before further analyses (see Fig. S3). A nonmetric multidimensional scaling (NMDS) ordination (stress = 0.15) showed a clustering of fungal communities by geographical location, apparently little affected by other factors except for plant host in the samples from Germany (SW_H and SW_G; Fig. 1b). Variance partitioning confirmed this by indicating that location exclusively explained nearly 15% of all variance, with all other factors only explaining a joint 4.3%. When we analyzed samples from every site individually, host species explained a significant 7 to 32% of local variances (Table 1), indicating important differences in the recruitment of soil fungi from local species pools. The nutrient conditions had only marginal effects on the assembly of communities (Table 1).

**TABLE 1:** Variance partition of fungal communities in roots of bait plants

Site^*a*^	Host			Nutrients			Host:nutrients	
	*F*_1_	*R* ^2^_adj_	*P*_adj_	*F*_1_	*R* ^2^_adj_	*P*_adj_	*F*_1_	*P*_adj_
AL_H	**3.2**	**0.11**	**0.001**	1.2	0.01	0.190	1.2	0.340
AL_G	**3.4**	**0.11**	**0.001**	**2.1**	**0.05**	**0.030**	1.8	0.080
CB_H	**3.6**	**0.13**	**0.001**	1.5	0.03	0.133	1.1	0.359
CB_G	**3.5**	**0.16**	**0.001**	0.9	0.00	0.533	0.9	0.603
SW_H	**6.2**	**0.26**	**0.001**	1.4	0.02	0.190	1.2	0.340
SW_G	**9.2**	**0.32**	**0.001**	1.1	0.00	0.302	1.6	0.288
VE_H	**2.6**	**0.08**	**0.001**	1.1	0.01	0.302	1.2	0.340
VE_G	**2.2**	**0.07**	**0.001**	1.4	0.02	0.165	**1.7**	**0.030**
FU_H	**3.2**	**0.16**	**0.002**	**2.3**	**0.09**	**0.020**	0.7	0.722
FU_G	**2.6**	**0.10**	**0.017**	1.7	0.04	0.190	1.7	0.288

a Abbreviations for sampling sites: AL, Los Alcornocales Natural Park (Spain); CB, Cabañeros National Park (Spain); SW, Schwarzwald/Black Forest National Park (Germany); VE, Mossel, Veluwe region (The Netherlands); FU, Fulufjället National Park (Sweden). Suffixes “_H” and “_G” denote heathland and grassland sites, respectively. Significant values (*P*_adj_ < 0.05) are shown in bold face. See Table S1 at https://doi.org/10.6084/m9.figshare.14222264 for more details.

10.1128/msystems.00304-22.4 FIG S3 Number of reads per sample obtained by Illumina MiSeq amplicon sequencing of fungi in roots of plant baits. Values correspond to log_10_(*x *+* *1)-transformed read numbers obtained for individual bait plants inoculated with nonsterilized (NS) soil, or from pooled (*n *=* *5) samples of plants inoculated with sterilized (S) soil. The box-and-whisker plots show the data distributions, and points indicate the individual values. Download FIG S3, PDF file, 0.03 MB. Copyright © 2022 Maciá-Vicente et al. 2022 Maciá-Vicente et al. https://creativecommons.org/licenses/by/4.0/ This content is distributed under the terms of the Creative Commons Attribution 4.0 International license.

Comparisons of the fungal communities recruited in the bait bioassays with those naturally occurring in the original habitats (25) showed that the former represented only a small subset of the latter (see Fig. S4). Although every sample yielded a number of OTUs not previously detected in the original sites, the proportions of OTUs shared with the respective natural communities (48.7 to 74.6%; see Fig. S4) were significantly higher than those obtained by random comparisons across samples (6.2 to 38.1%, *P < *0.001; see Table S5 at https://doi.org/10.6084/m9.figshare.14222264), thus confirming that the soil inocula were the sources of fungi colonizing the root baits. Compositionally, fungal OTUs in root baits and natural sites belonged to a similar set of dominant fungal orders (Fig. 1c), with eight orders shared out of the 10 most frequent per data set. Nevertheless, the proportions in the abundance of these orders varied between baits and original sites across samples, with the only exception of the *Helotiales* that markedly dominated most communities irrespective of their origin (Fig. 1c).

We isolated 442 fungal cultures from arabidopsis and barley roots, which were first grouped into 210 morphotypes, and subsequently into 152 OTUs by ITS and LSU sequencing (see Fig. S5; see Table S3 at https://doi.org/10.6084/m9.figshare.14222264). The distribution of fungal orders among isolates markedly differed from that obtained by sequencing, with an underrepresentation of *Helotiales* and *Sordariales*, and an enrichment of *Hypocreales*, *Pleosporales*, and *Eurotiales* (Fig. 1d) that reflects known biases of culturing methods (33). Nearly 80% of cultured OTUs were site specific, thus indicating a strong fungal endemism within sampling sites.

10.1128/msystems.00304-22.6 FIG S5 Phylogenetic tree of 152 OTUs representing all fungal isolates obtained. The tree is based on a maximum-likelihood analysis of a concatenated alignment of ITS and LSU rDNA region sequences. Download FIG S5, PDF file, 0.02 MB. Copyright © 2022 Maciá-Vicente et al. 2022 Maciá-Vicente et al. https://creativecommons.org/licenses/by/4.0/ This content is distributed under the terms of the Creative Commons Attribution 4.0 International license.

### Influence of organic nutrient sources on plant-fungus interactions.

**FIG 2:**
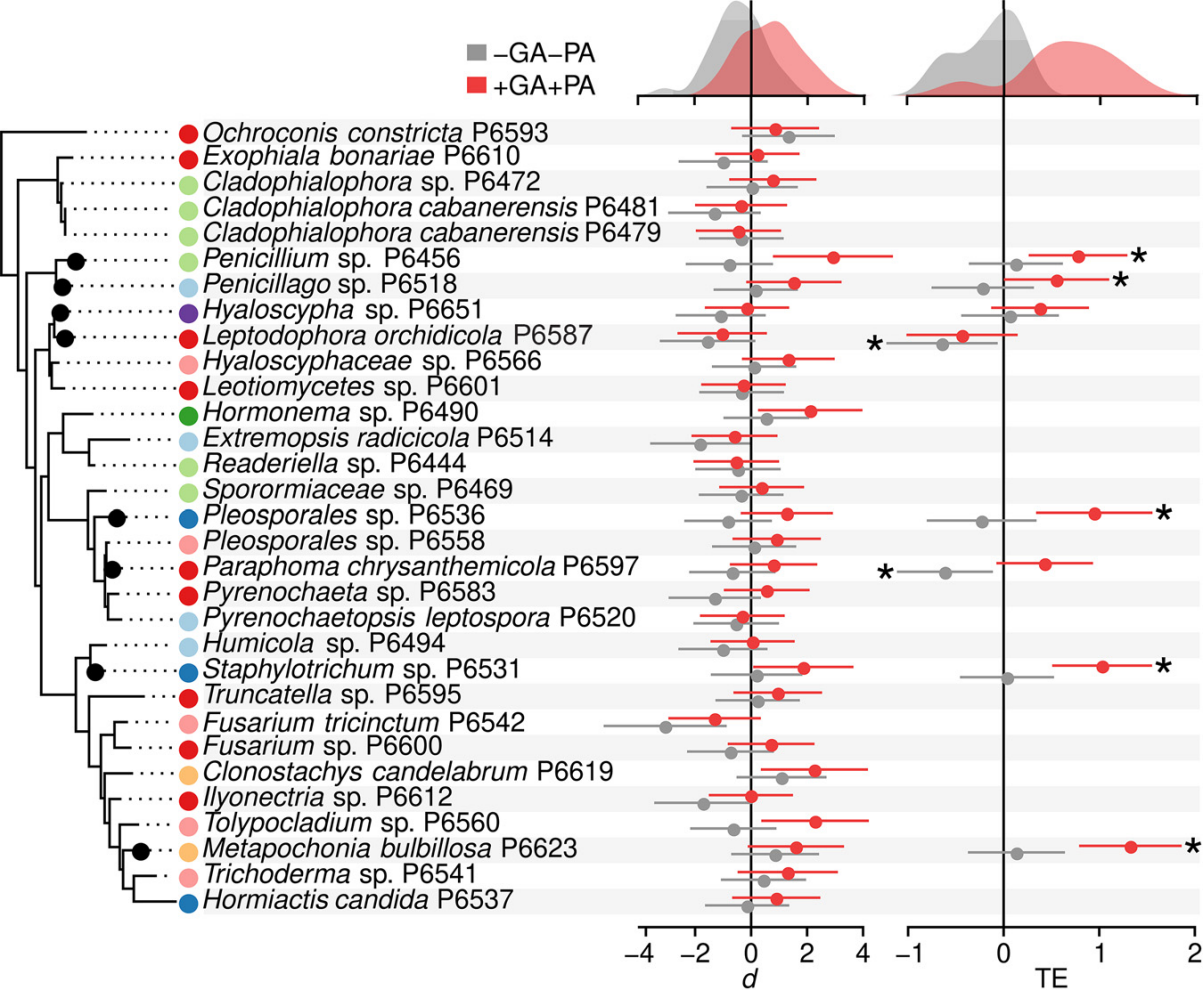
Effects of fungi on Arabidopsis thaliana growth under the absence or presence of organic nutrient sources. The phylogenetic tree to the left shows the 31 fungal strains included in plant inoculation bioassays. A subset of eight strains included in a second round of assays is highlighted by circles in the tree tips. Colored bullets next to the strain names represent the sites of origin, with colors following the color key in Fig. 1a. The plot in the middle shows the effects on *A. thaliana* growth of inoculation with the 31 strains in the absence (gray) or presence (red) of glutamic acid (GA) and phytic acid (PA), as organic sources of N and P, respectively. Points represent effect sizes (Cohen’s *d*) of fungal treatments respect to the uninoculated controls, and bars represent 95% confidence intervals. The plot rightward shows the effects on plant growth of fungal and nutrient treatments in repetitions of the experiment using a selection of eight fungal strains. In this case, points and bars represent the estimated treatment effects (TE) and confidence intervals calculated by a cumulative meta-analysis, by pooling effect size results from four experimental repetitions. Asterisks next to bars indicate significant differences respect to the uninoculated controls in tests of overall effect (*P < *0.05). Density plots on top of each plot represent the distribution of effect size values for all fungi under presence or absence of GA and PA.

We tested a selection of 31 fungal isolates, representing diverse lineages and geographical/ecological origins (Fig. 2; see Table S3 at https://doi.org/10.6084/m9.figshare.14222264), for their effects on growth of arabidopsis both under absence and presence of GA and PA. All isolates were reisolated upon plating surface-disinfected roots samples on growth media, suggesting effective endophytic colonization of roots. Different isolates caused a range of effects on plant growth compared to uninoculated controls, although these effects were not phylogenetically conserved (Fig. 2). Despite the large variability of plant effects across fungi, the amendment with nutrients resulted in a consistent increase in plant growth effect sizes across fungal treatments (Fig. 2): whereas without nutrient addition fungal inoculation had an overall negative effect on plant growth (median Cohen’s *d = *–0.47), the addition of GA and PA led to a significant (Wilcoxon test; *W *=* *205, *P < *0.001) shift toward a positive effect (median *d *=* *0.78). Three isolates, *Extremopsis radicicola* P6514, Fusarium *tricinctum* P6542, and *Ilyonectria* sp. P6612, significantly decreased plant growth under no nutrient amendment based on analysis of 95% confidence intervals, whereas five isolates, *Clonostachys candelabrum* P6619, *Tolypocladium* sp. P6560, *Hormonema* sp. P6490, *Penicillium* sp. P6456, and *Staphylotrichum* sp. P6531, significantly enhanced growth under GA/PA amendment. The effects on plant growth were measurable both by fresh biomass and canopy area (Fig. 2; see also Fig. S2) and were seldom accompanied by disease symptoms, with the exception of *F. tricinctum* P6542.

10.1128/msystems.00304-22.3 FIG S2 Comparison between Arabidopsis thaliana shoot fresh weight and total area measurements. (a) Points correspond to measurements per plant averaged for individual vessels (*n *=* *3), corresponding to the first plant inoculation bioassay, in which 31 fungal isolates were tested. The line represents a linear regression between both measures. (b) Photographs showing examples of the detection of *A. thaliana* shoot area. Images to the left show the original photographs, and images to the right show the shoot areas after detection of greens. Download FIG S2, PDF file, 0.1 MB. Copyright © 2022 Maciá-Vicente et al. 2022 Maciá-Vicente et al. https://creativecommons.org/licenses/by/4.0/ This content is distributed under the terms of the Creative Commons Attribution 4.0 International license.

A second round of assays included a subset of eight isolates and entailed a larger sample size (*n *=* *10) and up to four experimental repetitions per isolate (see Table S4 at https://doi.org/10.6084/m9.figshare.14222264). The isolates were selected to represent the different effects on plant growth observed in the previous experiment, as well as the main fungal lineages found in roots. The results confirmed the overall increase in plant growth effect sizes caused by fungal inoculation under nutrient amendments (*W *=* *6, *P = *0.005; Fig. 2). This increase was consistent across four independent repetitions of the experiment (see Table S4 at https://doi.org/10.6084/m9.figshare.14222264) and significant (*P < *0.05) in five isolates scattered across the fungal phylogeny, based on a meta-analysis of all repetitions (Fig. 2). Of the remaining three isolates, two caused significant reductions of plant growth without nutrient amendment, but these effects became neutral with amendment (Fig. 2).

The eight isolates tested accumulated more mycelial biomass in pure cultures when amended with either GA or PA compared to unamended medium, indicating a capacity to assimilate both compounds (see Fig. S6). This was also evident by fungal mycelium sometimes becoming visible on the surface of the growth substratum (see Fig. S6). We aimed at evaluating whether the fungal break-down of organic nutrients resulted in an enhanced acquisition of N and P by arabidopsis, thereby explaining the increases in plant growth. However, the overall low biomass of plants in this experiment precluded measurements of N contents in plant tissues by analytical methods available to us. Measurement of total P in shoots via ICP-OES indicated a significant increase in the assimilation of this element under nutrient amendments (two-way analysis of variance [ANOVA]; *F_1_* = 61, *P < *0.001; see Fig. S7). However, although there were significant differences in overall P concentration across fungal treatments (*F_8_* = 2.8, *P < *0.014), none of them implied differences with the uninoculated controls (Dunnett’s test, *P > *0.16; see Fig. S7). We obtained an analogous result when comparing total P content in shoots (see Fig. S7).

10.1128/msystems.00304-22.7 FIG S6 Growth of selected fungal strains on presence of glutamic acid (GA) and phytic acid (PA). (a) Dry weight of the eight fungal strains used in plant inoculation bioassays grown in either absence or in the presence of GA or PA. (b) Photographs showing examples of fungal mycelium (arrowheads) visible on the surface of the substratum of plant inoculation bioassays amended with GA and PA. Download FIG S6, PDF file, 0.05 MB. Copyright © 2022 Maciá-Vicente et al. 2022 Maciá-Vicente et al. https://creativecommons.org/licenses/by/4.0/ This content is distributed under the terms of the Creative Commons Attribution 4.0 International license.

10.1128/msystems.00304-22.8 FIG S7 Phosphorus concentration (a) and total content (b) in shoots of Arabidopsis thaliana plants calculated by inductively coupled plasma-optical emission-spectroscopy (ICP-OES), using specimens from one experimental repetition. Bars represent the mean from three pooled replicates, and error bars indicate the standard errors. In both cases, two-way ANOVAs indicated significant differences across fungal (*P < *0.001) and nutrient (*P < *0.01) treatments, but Dunnett’s *post hoc* tests showed no differences between fungal treatments and uninoculated controls (*P > *0.05). Download FIG S7, PDF file, 0.03 MB. Copyright © 2022 Maciá-Vicente et al. 2022 Maciá-Vicente et al. https://creativecommons.org/licenses/by/4.0/ This content is distributed under the terms of the Creative Commons Attribution 4.0 International license.

### Transcriptomic responses to fungal colonization.

**FIG 3:**
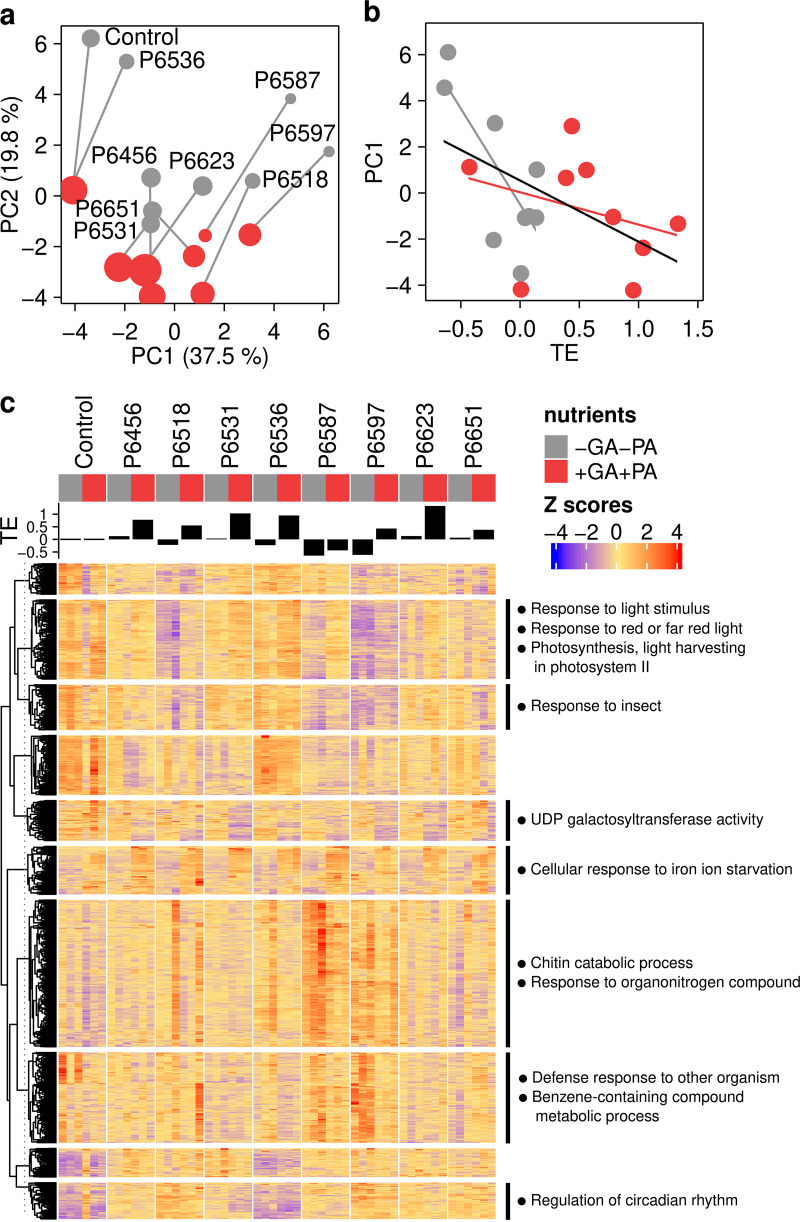
Transcriptome profiles of Arabidopsis thaliana in response to fungal inoculation and nutrient amendments. (a) Principal-component analysis (PCA) ordination of sample distances between *A. thaliana* shoot samples (median of three replicates) in response to colonization by different fungal strains, and to amendments with glutamic acid (GA) and phytic acid (PA). For every fungal treatment, samples of plants unamended (gray) and amended (red) with GA and PA are connected by a gray line. Point sizes are relative to plant growth effect sizes (estimated treatment effects, TE), as shown in Fig. 2c. (b) Correlation between the first axis (PC1) of PCA in panel a and TE values. Lines represent linear model regressions for the full data set (black) and for unamended and amended samples individually, included for illustrative purposes only. (c) Expression patterns and Gene Ontology (GO) annotation of *A. thaliana* transcriptomes. The heatmap shows median-centered *z*-scores for 1,596 differentially expressed genes across samples, arranged by *k*-means clustering. Black bars on top of the heatmap represent plant growth effect sizes (TE). Next to each cluster, significantly regulated GO terms (*P < *0.05) are indicated.

We detected 21,767 genes after mapping expression reads data to the arabidopsis genome, 1,596 of which were differentially expressed in at least one fungal or nutrient treatment compared to uninoculated, unamended controls. A principal-component analysis (PCA) of sample distances based on differentially expressed genes showed that the fungal effect on plant growth was the largest determinant of dissimilarity (Fig. 3a), with the first axis (PC1, 37% of variance) correlated with plant growth effect sizes (Pearson’s *r *= –0.5, *P = *0.029), particularly when no amendments with GA/PA were applied (*r *= –0.8, *P = *0.014; Fig. 3b). Nutrient treatment followed in determining sample dissimilarities, mainly compiled in the PCA’s second axis (PC2, 19.8% of variance). Altogether, this suggests that fungi with similar effects on plant growth also elicit similar gene expression patterns in the host.

We explored whether the differential expression of genes implicated in the plant’s response to N and P starvation (i.e., with GO terms including the strings “nitrogen starvation,” “response to nitrogen,” or “phosphate starvation”) were associated with the growth responses to treatments by correlating their median-centered *z*-scores with PC1 (see Fig. S8). If growth promotion is due to alleviation of N and/or P starvation under GA/PA amendment, we would expect significant correlations between the expression of these genes and PC1 under amendment conditions only. We identified two differentially expressed genes associated with N starvation responses and 27 with P responses (see Fig. S8), of the total 23 and 28 genes respectively associated with these functions in the arabidopsis genome, thus indicating a major response to P but not to N limitation. Although multiple genes showed strong correlations with PC1 (|*r*| > 0.5), the statistical power was inadequate to detect significant differences, or correlations were similar for unamended and amended treatments (see Fig. S8). The expression patterns for the individual genes selected also did not show a common pattern of variation indicative of such a response (see Fig. S9).

10.1128/msystems.00304-22.9 FIG S8 Pearson’s correlation between expression patterns for selected genes, implicated in Arabidopsis thaliana’s response to N and/or P limitation, and the fungal effects on plant growth, as summarized by the PC1 axis of Fig. 1a. Bars represent Pearson’s *r* for either GA and PA unamended and amended treatments, and asterisks indicate significant correlations at *P < *0.05, after being adjusted for multiple comparisons by the Benjamini-Hochberg method. Download FIG S8, PDF file, 0.03 MB. Copyright © 2022 Maciá-Vicente et al. 2022 Maciá-Vicente et al. https://creativecommons.org/licenses/by/4.0/ This content is distributed under the terms of the Creative Commons Attribution 4.0 International license.

10.1128/msystems.00304-22.10 FIG S9 Expression patterns of Arabidopsis thaliana genes implicated in the plant’s response to N or P starvation, significantly regulated upon fungal inoculation and/or amendment with organic sources of N and P. For each gene, the individual values of median-centered *z*-scores per sample are shown. Download FIG S9, PDF file, 0.06 MB. Copyright © 2022 Maciá-Vicente et al. 2022 Maciá-Vicente et al. https://creativecommons.org/licenses/by/4.0/ This content is distributed under the terms of the Creative Commons Attribution 4.0 International license.

Aiming to find alternative mechanisms driving the plant growth promotion triggered by fungi in nutrient-amended plants, we performed an untargeted analysis of transcripts by grouping the differentially expressed genes into 10 clusters based on expression patterns and performing GO-term enrichment analysis (Fig. 3c; see Table S6 at https://doi.org/10.6084/m9.figshare.14222264). This revealed that most genes clustered into sets whose expression varied in response to fungal colonization by different strains and, to a lesser extent, to nutrient conditions (Fig. 3c). Only one cluster showed a pattern exclusively influenced by the nutrient condition, with a consistent enrichment under GA/PA amendment of genes encoding transcription factors involved in the cellular response to iron ion starvation (Fig. 3c; see Table S6 at https://doi.org/10.6084/m9.figshare.14222264). A second major gene set was strongly enriched for genes in photosynthesis processes and light reception (Fig. 3c), which became downregulated in most fungal treatments, particularly in those with more detrimental effects on plant growth, and in the absence of organic nutrients (Fig. 3c). Most other clusters of significantly regulated genes comprised gene functions implicated in the plant’s defense response against colonization, with genes (i) encoding the indole glucosinolate metabolism (included in the main term “response to insect,” Fig. 3c; see Table S6 at https://doi.org/10.6084/m9.figshare.14222264), or mediating (ii) the systemic acquired resistance (SAR) dependent upon salicylic acid accumulation (“defense response to other organism” and “benzene-containing compound metabolic process”), triggered by both biotic and abiotic stimuli (34), and (iii) the plant’s response to chitin (“response to organonitrogen compound” and “chitin catabolic process”). The latter two clusters were especially upregulated in nutrient-unamended plants colonized by *Leptodophora orchidicola* P6587 and *Paraphoma chrysanthemicola* P6597, which showed significantly impaired growth (Fig. 2).

## DISCUSSION

Our results show a generalized growth promotion of arabidopsis by several root-colonizing fungi, which only takes place when plants are subject to nutrient limitation but supplied with organic sources of N and P that are common in soils but inefficiently taken up by most plants. Although such an effect is suggestive of a fungal facilitation of the host’s uptake of nutrients, e.g., via mineralization or direct transport of nutrients through hyphal networks, we could not verify this by direct nutrient measurements or specific changes in the gene expression profiles of the plants. Instead, plant growth responses to fungal colonization mainly entailed the regulation of genes implicated in host immunity and defense against microbes and photosynthetic efficiency, among other genes without well-defined molecular functions. Notably, phylogenetically and ecologically diverse fungi elicited similar phenotypes and transcriptome responses in arabidopsis, suggesting a common plant reaction to different root-colonizing symbionts.

In contrast to what we hypothesized, the recruitment of soil fungi by roots in plant bait bioassays was barely affected by nutrient availability. The consistent reduction in above-ground plant biomass under low nutrient levels, particularly in arabidopsis, indicates a growth limitation that is usually accompanied by changes in root morphology, physiology, and metabolism (35, 36) and that would expectably lead to a differential selection of microbes with distinct functional traits through the modulation of root niches. However, our overall results show that fungal communities in roots are resilient to changes in the nutritional status of the hosts and/or the nutrient composition of the substratum, irrespective of host plant identity. This is in line with results that showed little effects of soil P content on the composition of the arabidopsis root mycobiome (37), although another study indicated a somewhat larger effect of P amendments on fungal community assembly, explaining up to 20% of community variation (23). At any rate, if recruitment of soil fungi is selective for particular genotypes that provide stress alleviation—as is known to occur in arbuscular mycorrhizas (22, 38)—an enrichment of particular fungal lineages rather than community-wide shifts is to be expected. However, because fungal traits pivotal for the interaction with plants (either mutualistic and detrimental) are often subject to strong selection pressure and thus vary between closely related species or even conspecific strains (17, 39, 40), such enrichment patterns may entail OTU-level changes across treatments that are harder to identify than more phylogenetically conserved traits. This is particularly true when considering the marked geographical endemism in root-associated fungi disclosed here and elsewhere (25, 41), in that similar niches may be occupied by distinct, locally adapted fungi.

We report here a consistent effect of fungal root colonization on plant response to organic nutrient amendments, resulting in enhanced (even if slight) biomass production compared to fungus-free plants. Whereas multiple studies have shown that nonmycorrhizal, root-colonizing fungi have overall negative impacts on plant growth, likely owing to the capture of plant resources by the fungal symbionts (42–46), fungus-driven growth promotions in response to organic amendments are also common in the literature. In particular, several works focusing on so-called dark septate endophytes (DSEs) (47)—a polyphyletic group of root-colonizing fungi with melanized hyphae—have shown a frequent plant growth promotion by these fungi in the presence of organic nutrient sources (12, 48–50). Here, we expand these observations and show that the effect is widespread across root-colonizing fungi, irrespective of their phylogenetic affiliation and of whether they express or not the morphology characteristic of DSEs. However, we could not draw a link between such growth promotion and a hypothetical fungal mobilization of nutrients from organic sources (51), since we did not find any evidence of an enhanced acquisition of N or P, neither by direct measurements (for P only) nor indirectly by transcriptome analysis. In this regard, although increases in shoot N and P contents have been described for DSE-inoculated plants amended with organic nutrients (50), these effects rely on total amounts that covary with concomitant increases in plant biomass and disappear when nutrient concentrations per unit of plant biomass are considered. Nevertheless, our inability to directly quantify N from plant tissues, as we did with P, is an important caveat of this study. Although the RNA-seq results did not show important plant transcriptome responses to N levels, we cannot entirely discount that the growth promotion caused by some isolates was accompanied by an increased uptake of N. Indeed, the ability of some fungi to assist in the assimilation of N from exogenous amino acids has been demonstrated elsewhere (14).

The similar response of arabidopsis to GA and PA amendment when colonized by most fungal strains, with a generalized increase in its growth with respect to noninoculated plants, suggests that the effect is driven by the host plant rather than by the fungi. This is reinforced by RNA-seq data showing a quantitative modulation of the plant transcriptome that is directly related with the fungal effects on growth; i.e., unrelated fungi triggering similar growth responses also induced similar rather than strain-specific gene expression patterns. Although the transcriptome profiles are not conclusive about the mechanisms underlying the plant growth responses observed, they seem to rule out a host rescue from N or P starvation and particularly discount a significant N stress response based on the reduced number of differentially expressed genes implicated in this function. Importantly, a trade-off between the arabidopsis P starvation and defense responses has been demonstrated (52, 53), with P-starved plants prioritizing investment of resources in coping with nutrient limitation over immunity toward microbial infections, a process that has been shown to be crucial to establishing beneficial associations with fungi (17, 54). Starving conditions could then favor the expression by particular fungal strains of specific beneficial traits upon root colonization, thereby explaining the differences in plant growth observed across fungi. In that case, elucidating the specific interaction mechanisms with every fungus would require further and individualized efforts. For example, the growth-promoting strains *Penicillium* sp. P6456 and *Metapochonia bulbillosa* P6623 have close relatives in *Penicillium simplicissimum* and *Metacordyceps chlamydosporia*, both of which have repeatedly shown host growth enhancement through unknown mechanisms, but always triggering a modulation of plant defense pathways (55–57).

An additional difficulty to elucidate the interplay between root-colonizing fungi and the host’s response to nutrient conditions originate from the inability to isolate purely nutritional effects of complex organic compounds from other bioactivities. Thus, besides constituting an important source of N in natural soils, GA can stimulate plant immune responses and thereby affect plant colonization by microorganisms (58, 59). As for PA, it can act as a strong chelator for cations such as iron (60), which could explain the marked and consistent response to iron starvation we detected in arabidopsis plants in the amendment treatment and that further interfered with the effects we sought to assess. Last, it is possible that our direct measurements of P assimilation into arabidopsis shoots were not sensitive enough to detect an effective increase in the uptake of this nutrient. Recent studies demonstrating the ability of root-colonizing fungi to translocate P to their hosts have relied in tracing of radioactive ^33^P (15, 16), which provides a high detection sensitivity. Nevertheless, Almario et al. (15) reported an increase in total shoot P content measured by inductively coupled plasma mass spectrometry, alongside the fungal transfer of ^33^P to the plant, indicating that conventional analytical tools, such as the one we used, can also detect contributions of fungi to plant nutrition.

In conclusion, we show that root-colonizing fungi can promote host growth under nutrient-limiting conditions, but we could not trace the mechanisms underlying this effect. Plant responses to root colonization by phylogenetically and ecologically diverse fungi may primarily result from the interplay between the plant immune system and the local environment. The common effects of different nonmycorrhizal fungi on plant performance suggest a functional convergence in root-associated mycobiomes that are strongly structured across space and habitats.

## MATERIALS AND METHODS

### Soil samplings.

We collected soil samples in 2018 from adjacent seminatural heathlands and grasslands at five geographical locations along a latitudinal gradient in Western Europe, spanning from southern Spain to mid Sweden (Fig. 1a). The description of the sites and the procedures for the collection of samples are provided in Maciá-Vicente and Popa (25). In brief, we selected the locations within protected natural areas, after obtaining collection permits from the relevant local authorities, to ensure as little anthropogenic disturbance as possible. In each location, we sampled at two sites, in one heathland and one grassland, separated less than 2 km from one another. The two habitat types were selected because, when undisturbed, they impose different types of nutrient limitation (61). At each site, we defined a 4 × 4-m plot from which we collected subsamples of soil associated with the roots of up to 16 individuals from each of two locally dominant plant species, mainly a monocot and a eudicot (see Table S1 at https://doi.org/10.6084/m9.figshare.14222264). We collected the soil subsamples by uprooting the entire plants, or large portions of their roots, and taking ~20 cm^3^ of the associated soil from each individual. All soil subsamples from each site were pooled and processed in the laboratory within 48 h after their collection. Details of the sampling sites, plant species, and soil characteristics are provided in Table S1 at https://doi.org/10.6084/m9.figshare.14222264.

### Baits for root-associated fungi.

We prepared two 1% (wt/vol) suspensions of each soil sample in filter-sterilized phosphate-buffered saline solution (130 mM NaCl, 7 mM Na_2_HPO_4_, 3 mM NaH_2_PO_4_, 0.02% Tween 20 [pH 7.0]). One of the two soil suspensions was sterilized by autoclaving at 121°C for 20 min to serve as a negative inoculation control. Arabidopsis thaliana ecotype Col-0 (arabidopsis) and *Hordeum vulgare* cv. Barke (barley) seeds were sterilized, pregerminated, and grown under gnotobiotic conditions on clay granules in Magenta vessels (77-mm width × 77-mm length × 97-mm height; GA-7-3, Sigma, St. Louis, MO) or on vermiculite in glass tubes (25-mm diameter × 150-mm length; C-5916, Sigma), respectively, following procedures described elsewhere (62, 63). Containers for each plant species were irrigated at the beginning of the experiment with either 0.5× or 0.05× Hoagland’s nutrient solution (H2395, Sigma; see Table S2 at https://doi.org/10.6084/m9.figshare.14222264) to subject plants to conditions of nutrient availability or limitation, respectively. Upon planting the seedlings, we inoculated each container with 2 mL of either sterilized or nonsterilized soil suspensions, by spreading them evenly over the surface of the substratum. Every treatment comprised five replicates, each consisting of a container with three plants in the case of arabidopsis, or one barley plant. Plants were maintained at 23°C under a 12 h:12 h (light:dark, 80 μmol m^−1^ s^−1^) photoperiod in plant growth chambers (KBW400; Binder, Tuttlingen, Germany).

We harvested the plants 30 days after inoculation and measured the fresh weights of arabidopsis shoots (not roots, owing to the difficulty of separating fine roots from clay granules), and barley shoots and roots. We thoroughly washed the roots from every container and split them into two halves; one half was immediately used for fungal isolation, and the other one was frozen at −76°C until DNA extractions.

### Isolation and characterization of fungi from roots.

We surface-disinfected the roots by first washing them with 0.5% (vol/vol) NaOCl for 1 min and then rinsing them three times with autoclaved distilled water. We individually macerated a 10 cm-long root piece from every sample in 1 mL of 0.05% (wt/vol) agar using a Retsch MM 200 mixer mill (Retsch GmbH, Haan, Germany), and then plated 200 μL of the root suspension on a 140 mm-diameter petri dish containing 0.5% (wt/vol) malt extract agar (Applichem, Darmstadt, Germany) supplemented with 0.1% (vol/vol) Triton X-100 (Sigma) and 0.5 g L^−1^ chloramphenicol (Applichem). We monitored the plates for up to 2 months and characterized fungal colonies by their rough morphology as they appeared. We isolated representative colonies from every morphology and treatment on potato dextrose agar (Applichem), grouped them further into morphotypes, and characterized them by sequencing both the internal transcribed spacer (ITS) and the large subunit (LSU) regions of the rDNA. We used the sequence data to group isolates into phylotypes and to build a phylogenetic tree where all these are represented. The detailed procedures used to characterize the fungi are provided in Text S1, and data on all isolates are given in Table S3 at https://doi.org/10.6084/m9.figshare.14222264. Where necessary, we obtained authorization from National Focal Points of the Convention on Biological Diversity for work with the fungal isolates, as stipulated by the Nagoya Protocol.

10.1128/msystems.00304-22.1 TEXT S1 Detailed methodology for the molecular characterization of root-associated fungal isolates, preprocess and analysis of Illumina MiSeq datasets, inoculation of Arabidopsis thaliana with fungal isolates, measurement of *A. thaliana* total shoot area, and total RNA extraction from *A. thaliana* shoots. Download Text S1, DOCX file, 0.04 MB. Copyright © 2022 Maciá-Vicente et al. 2022 Maciá-Vicente et al. https://creativecommons.org/licenses/by/4.0/ This content is distributed under the terms of the Creative Commons Attribution 4.0 International license.

### Fungal amplicon sequencing.

We extracted total DNA from roots and used it for Illumina MiSeq sequencing of fungal ITS amplicons using the primers ITS1F and ITS2 (64), modified as reported previously (65). Roots from replicates in the negative controls were pooled together treatment-wise before processing. The preparation of samples, the preprocess of sequence reads, and their grouping into OTUs, followed procedures described previously (25). We excluded all OTUs present in the negative inoculation controls and then compared community composition across the inoculated root samples using NMDS based on Bray-Curtis dissimilarities. We then used variation partitioning analysis to investigate the influence of the factors geographical location, habitat type (heathland versus grassland), host species, and nutrient condition on community assembly. To evaluate whether the fungal diversity included in our data set is representative of that in natural conditions, we compared our list of OTUs with that of Maciá-Vicente and Popa (25), including the fungal communities in roots and soil at the original sampling sites. Details of the preprocessing and analysis of read data are provided in Text S1.

### Bioassays to test the influence of organic nutrient sources on plant-fungus interactions.

We tested for the influence of the amendment with organic sources of N and P on the interactions between root-associated fungi and arabidopsis, using a plant inoculation bioassay similar to that described above. In this case, the vessels were watered with 0.05× Hoagland’s solution to keep conditions of nutrient starvation, and plants were inoculated with hyphal suspensions of individual fungal isolates 1 day after sowing, or with sterilized distilled water in uninoculated controls (see Text S1). Four days after inoculation, we subjected the plants to either of two nutrient treatments: one without amendment of organic nutrients (–GA–PA), by adding 4 mL of sterilized distilled water, and one with amendment (+GA+PA), by adding 4 mL of a 202.5 μM GA, 2.25 μM PA (pH 5.2) solution. We calculated the quantities of GA and PA to equal the difference in N and P atoms between irrigations with 0.5× and 0.05× Hoagland’s that had previously shown to significantly affect plant growth (see Fig. S1). We harvested the plants 30 days after inoculation and measured shoot fresh weight and/or total area (see Text S1). Because both measurements showed comparable results (see Fig. S2), we relied on total shoot area throughout the study. We calculated the effect sizes of measurements for fungus-inoculated versus uninoculated plants within each nutrition treatment, using the Cohen’s *d* statistic (66) as described previously (42).

In a first set of assays, we screened a selection of 31 isolates representative of the main fungal lineages found (see Table S3 at https://doi.org/10.6084/m9.figshare.14222264). In this case, the treatments included five replicates, each consisting of a vessel with three arabidopsis plants in each, and the screening was performed in two batches in which every fungal treatment was compared to a respective uninoculated control. At the end of the experiment, we confirmed root colonization by the inoculated fungi by plating surface-disinfected roots from every treatment. Root disinfection was done by washing roots with 0.5% sodium hypochlorite for 1 min, followed by three rinses with sterilized water. In a second set of assays, we further tested a subset of eight isolates using 10 replicates per treatment (see Table S3 at https://doi.org/10.6084/m9.figshare.14222264). We repeated the latter experiment four times in order to detect fungi with consistent effects on plant growth across repetitions, and due to the need to reach biomass enough for further tests, given the small size of plants as a result of the low nutrient conditions. In the second assay, we performed a cumulative meta-analysis of the effect sizes obtained across experiment repetitions using function *metagen*() of package META v4.15-1 (67) of r v3.6.3 (68) to look for robust statistical differences between treatments and uninoculated controls. In this case, we reported the effect sizes as estimated treatment effects (TE) with 95% confidence intervals (see Fig. 2 and 3).

### Phosphorus content in arabidopsis shoots.

We used the arabidopsis shoots in one repetition of the inoculation bioassays with eight isolates to measure the total P content in shoots (repetition 3, arbitrarily chosen due to similar results across experimental repetitions; see Table S4 at https://doi.org/10.6084/m9.figshare.14222264). Because of the small size of plants, shoots from all replicates within each treatment were pooled into three samples (*n *=* *10 plant individuals each), and these were then desiccated at 40°C. Approximately 5 mg from each sample were first digested with 69% HNO_3_ (Suprapur; Sigma) for 25 min, followed by addition of 1 mL of 30% H_2_O_2_ and sonication during 1 h. Finally, the samples were filtered through 0.45-μm-pore-size PTFE filters and analyzed by inductively coupled plasma-optical emission-spectroscopy (ICP-OES; Shimadzu ICPE-9000). Although we also attempted to quantify total N content in shoots, we did not manage to obtain enough dry biomass in pooled samples for measuring this element with standard methods available to us.

### RNA sequencing.

Shoots from another repetition of the arabidopsis inoculation bioassays (repetition 2, arbitrarily chosen; see Table S4 at https://doi.org/10.6084/m9.figshare.14222264) were used for gene expression analysis based on RNA sequencing (RNA-seq). Owing to the small size of plants, we pooled replicates within treatments into three samples (*n *=* *10) to reach enough biomass for RNA extraction. We deep-froze shoots in liquid nitrogen immediately after harvesting, and then purified RNA using a custom protocol (see Text S1). RNA-seq libraries were prepared from 1 μg of total RNA and pair-end sequenced (150-bp reads) using the Illumina HiSeq2500 sequencing system by Novogene Co. (Beijing, China).

We mapped high-quality reads to the arabidopsis reference genome sequence of The Arabidopsis Information Resource (TAIR) using the Hisat2 paired-end strategy with default settings (69) and applied featureCounts to count read hits on exons, scoring only unique mappings based on the *Arabidopsis* genome annotation file from Ensembl Plants (https://plants.ensembl.org). Differential gene expression analysis was performed in R using DESeq2 v1.26.0 (70), with which we extracted genes with significant expression differences in at least one treatment compared to the uninoculated and unamended controls, by using a cutoff of |log_2_FC| ≥ 1 and *P_adj_* < 0.01. We obtained scaled counts normalized to library size and transformed as median-centered *z*-scores, which were then used to conduct *k*-means clustering for all transcripts. We compared transcriptome profiles across samples using PCA, and then used heatmaps built with the ComplexHeatmap v2.2.0 package (71) to visualize differentially expressed transcripts and cluster results. Gene Ontology (GO)-term enrichment analysis, with annotations based on the Gene Ontology Consortium (72), were performed with Cytoscape v3.2.0 (73) using the plugins ClueGO v2.3.5 and CluePedia v1.3.5 (74, 75). Significantly enriched terms were determined at *P ≤ *0.05 using the hypergeometric test, with adjustment of *P* values using Holm-Bonferroni step-down correction. We specifically investigated genes with GO annotations indicating implication in responses to N and P starvation, by assessing the Pearson’s correlation of their expression patterns under either nutritional treatment with the first axis of the PCA built with transcriptome distances, which compiled most variance of plant growth in response to fungal inoculation.

### Data availability.

The MiSeq and RNA-seq data generated in this study have been deposited in the NCBI Sequence Read Archive under BioProject numbers PRJNA640064 and PRJNA706587. The ITS and LSU sequences obtained from fungal cultures are deposited in NCBI GenBank (see Table S3 at https://doi.org/10.6084/m9.figshare.14222264 for accession numbers). Representative fungal strains isolated in this study have been deposited in the CBS Culture Collection (Westerdijk Fungal Biodiversity Institute, Utrecht, The Netherlands).
