# Supplementary figures and images for: Nutrient Availability Does Not Affect Community Assembly in Root-Associated Fungi but Determines Fungal Effects on Plant Growth
Source: mSystems. 2022 Jun 13;7(3):e00304-22. doi: 10.1128/msystems.00304-22 (PMC9239174; doi:10.1128/msystems.00304-22)

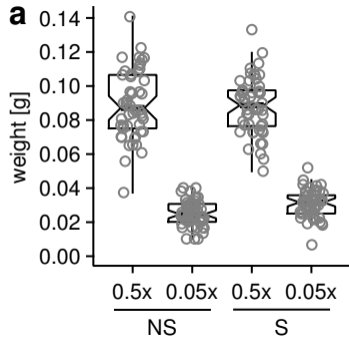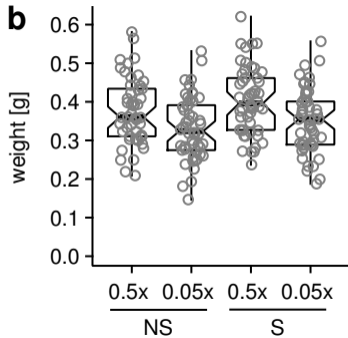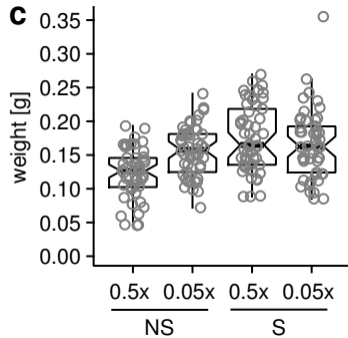

Supplement: FIG S1 [file msystems.00304-22-sf001.pdf]

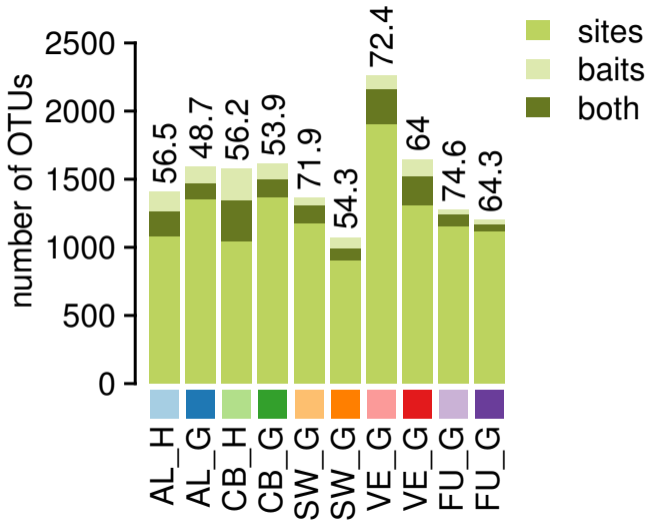

Supplement: FIG S4 [file msystems.00304-22-sf004.pdf]

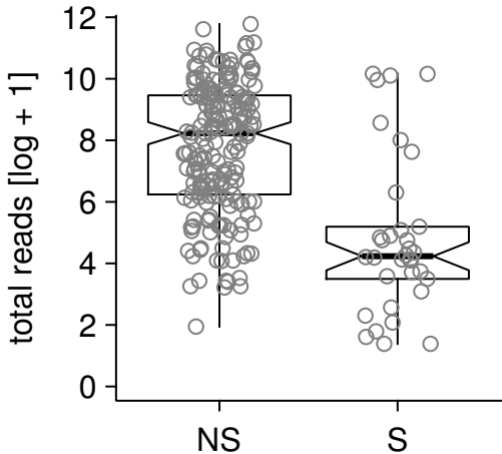

Supplement: FIG S3 [file msystems.00304-22-sf003.pdf]

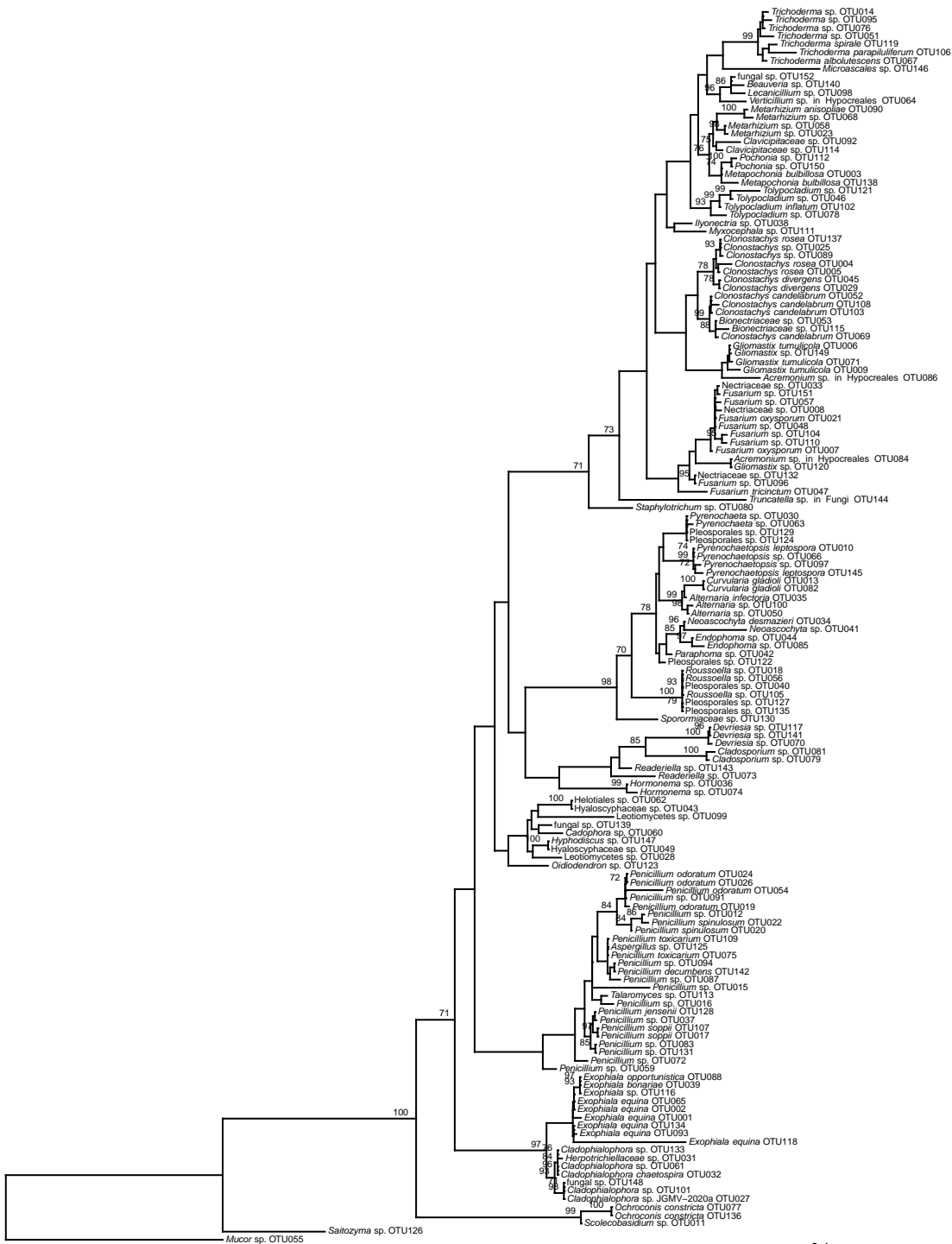

0.1

Supplement: FIG S5 [file msystems.00304-22-sf005.pdf]

**a**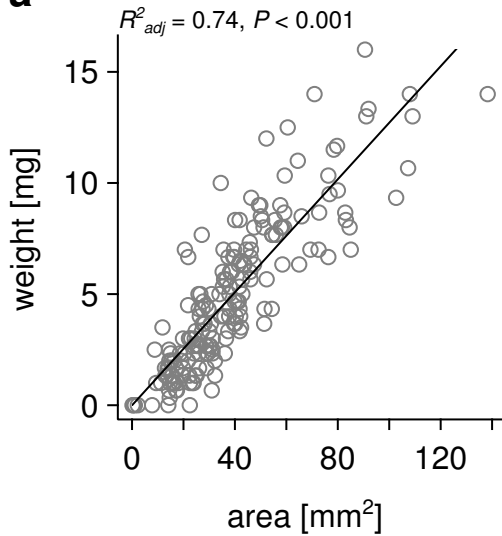**b**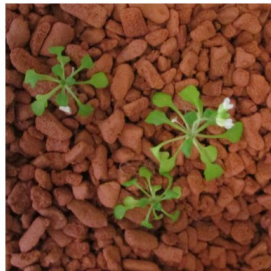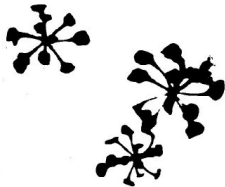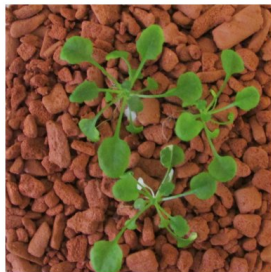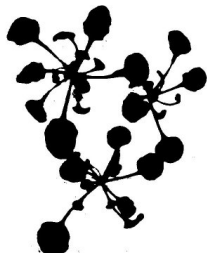

Supplement: FIG S2 [file msystems.00304-22-sf002.pdf]

**a**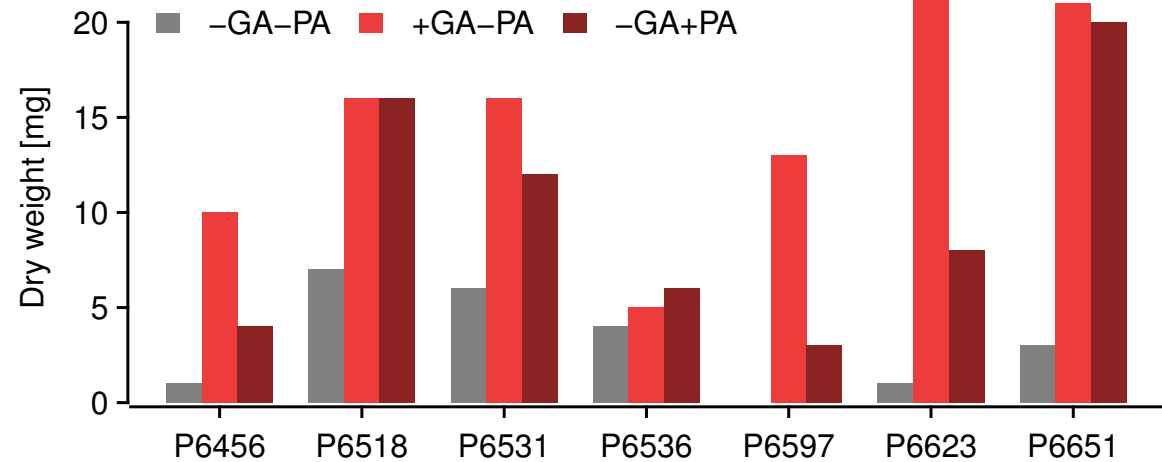**b**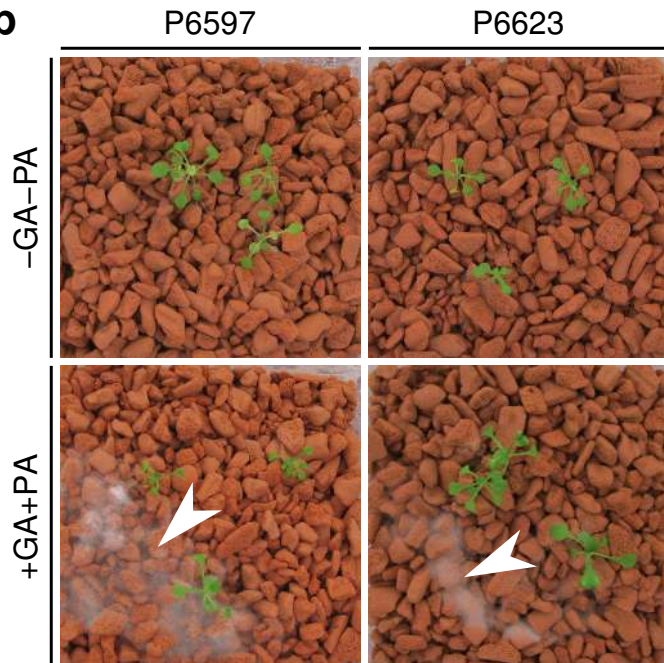

Supplement: FIG S6 [file msystems.00304-22-sf006.pdf]

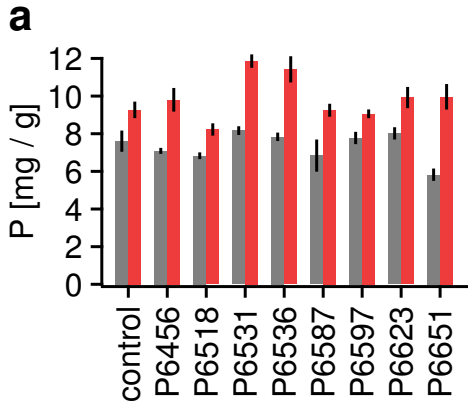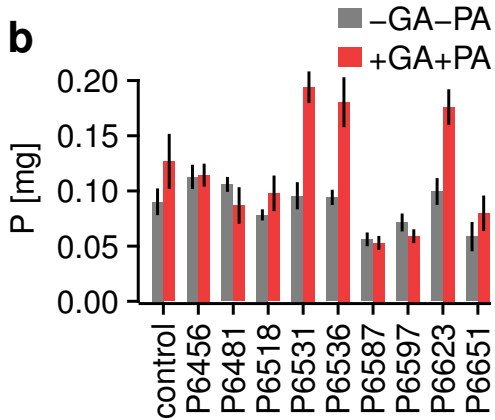

Supplement: FIG S7 [file msystems.00304-22-sf007.pdf]

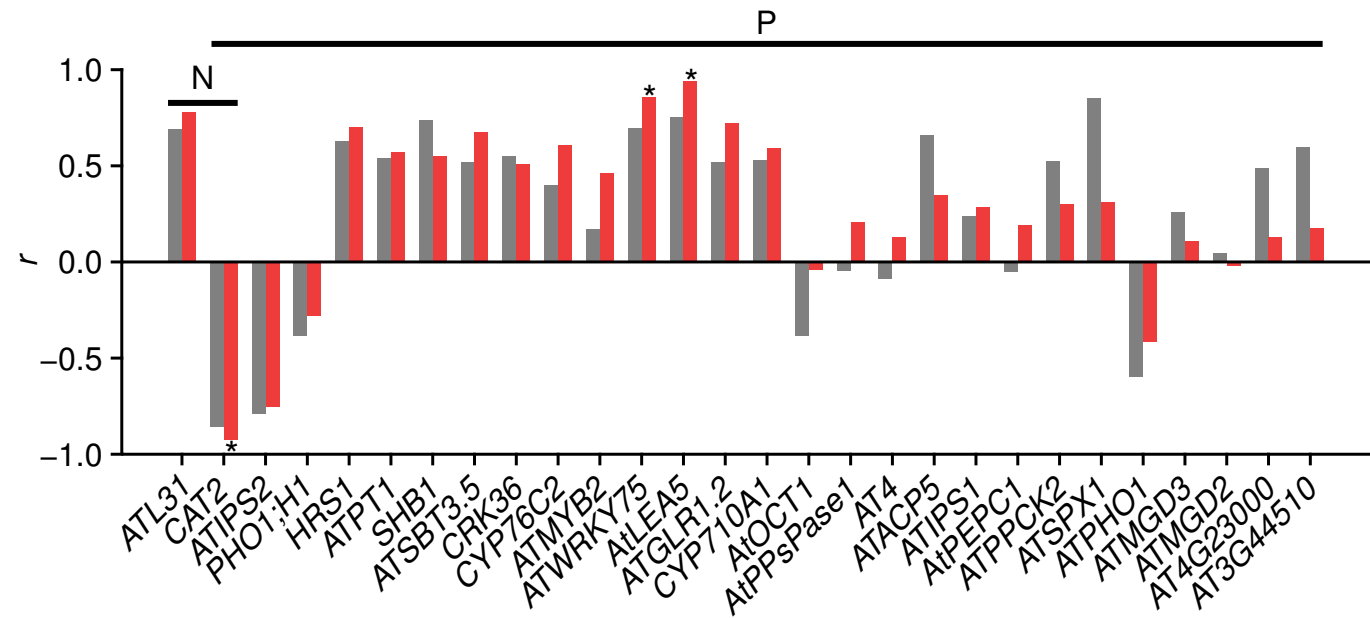

Supplement: FIG S8 [file msystems.00304-22-sf008.pdf]

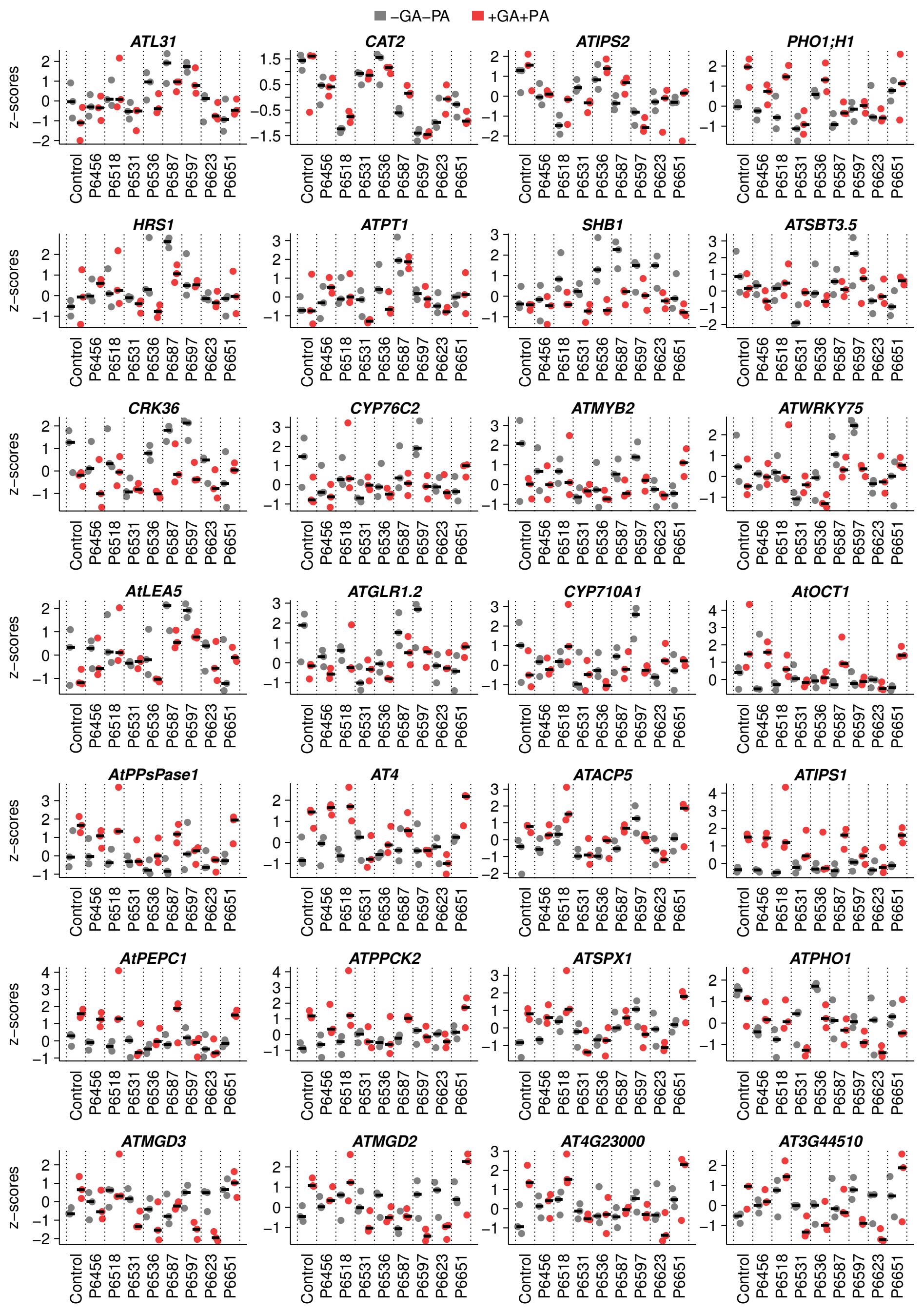

Supplement: FIG S9 [file msystems.00304-22-sf009.pdf]
