# Supplementary material for: Nutrient Availability Does Not Affect Community Assembly in Root-Associated Fungi but Determines Fungal Effects on Plant Growth
Source: mSystems. 2022 Jun 13;7(3):e00304-22. doi: 10.1128/msystems.00304-22 (PMC9239174; doi:10.1128/msystems.00304-22)
Supplement: TEXT S1 [file msystems.00304-22-t0001.docx]

**Supplementary Methods**

**Molecular characterization of root-associated fungal isolates.**

*Total DNA extraction from mycelium*

We collected mycelium from representative isolates by scrapping the surface of colonies in pure culture, and then resuspended it in 600 μl DNA extraction buffer (2% (w/v) cetyltrimethyl ammoium bromide, 100 mM Tris-HCl, 20 mM EDTA, 1.4 M NaCl, 1% (w/v) 40,000 polyvinyl pyrrolidone, 20 μg ml^-1^ RNAse A, pH 8.0) and froze it overnight at -20 °C. For DNA extraction, we thawed the mycelium and incubated it at 65 °C for 30–45 min, then added one volume of 24:1 chloroform:isoamyl alcohol and centrifuged at 17 000 *g* for 10 min. The DNA in the supernatant was precipitated in isopropanol at -20 °C for 20–40 min, pelleted by centrifugation at 11 000 *g* at 4 °C for 20 min, washed in ice-cold 70% ethanol, and resuspended in 50 μl nuclease-free water. We measured the quantity and quality of DNA using a Nanodrop spectrophotometer (NanoDrop products, Wilmington, DE, USA) and prepared aliquots of all samples at DNA concentrations of 10 ng μl^-1^.

*Amplification and sequencing of the ITS and LSU rDNA regions*

We simultaneously amplified the full rDNA internal transcribed spacer (ITS) region and a portion of the rDNA large subunit (LSU) using primers V9G and LR8 (2, 3). PCR reactions were done in 20 μl containing 10 ng of DNA template, 2 mM MgCl_2_, 0.5 mg ml^-1^ bovine serum albumin (New England Biolabs GmbH, Schwalbach, Germany), 0.2 mM of each dNTP (Bioline, Luckenwalde, Germany), 0.3 μM of each primer, and 0.5 units of Taq DNA polymerase (VWR International GmbH, Darmstadt, Germany). Amplifications were carried out in a Mastercycler pro thermal cycler (Eppendorf, Hamburg, Germany) at 94 °C for 4 min followed by 35 cycles of 94 °C for 30 s, 55 °C for 90 s, and 72 °C for 45 s, and a final step of 72 °C for 5 min. We used the amplicons to mostly sequence unidirectionally the ITS and LSU regions with primer ITS1F and LR0R (2, 4), respectively. Sequencings were performed by the sequencing laboratory of the Biodiversity and Climate Research Centre (Frankfurt am Main, Germany).

*Identification of isolates and grouping into phylotypes*

We assigned putative identifications to fungal isolates by comparing the ITS sequences against the UNITE database of reference fungal ITS sequences (5), using the Naïve Bayesian Classifier tool (6) available in mothur v1.39.5 (7). For a few isolates for which we failed to obtain ITS sequences, we manually assigned them to taxa by comparing their LSU sequences against the NCBI GenBank database using BLAST (8). We grouped isolates into operational taxonomic units (OTUs) based on pairwise similarities of ITS sequences calculated with the BLASTCLUST tool

(<ftp://ftp.ncbi.nih.gov/blast/documents/blastclust.html>) of the ncbi-blast package (Altschul *et al.* 1990), using a cut-off value of 97% sequence similarity. A consensus taxonomy was obtained for every phylotype using the *classify.otu* tool of mothur. Six isolates that yielded conflicting identifications by using their ITS and LSU sequences, and isolates for which ITS sequences could not be obtained, were excluded from the classification into OTUs.

*Phylogenetic analysis*

We built a phylogenetic tree based on concatenated ITS and LSU sequences, including representatives from all phylotypes obtained from fungal isolates. We aligned the datasets for each locus using mafft v7.271 (9) with the G-INS-i parameters, and then trimmed the alignments with Gblocks v0.91b (10) before concatenating them. We used the concatenated alignment to calculate a Maximum Likelihood phylogeny using RAxML v8.0 (11) with the general time reversible model of nucleotide substitution and the Γ model of rate heterogeneity (GTRGAMMA), allowing for different model parameter estimations per partition. Branch support was assessed by means of non-parametric bootstrapping with 1,000 replicates.

**Pre-process and analysis of Illumina MiSeq datasets.**

The pre-process and the initial analyses of the Illumina MiSeq sequence reads were performed together with the dataset presented in (1), and therefore follows the same pipeline. We assembled and processed the sequence reads using the DADA2 pipeline for quality filtering, dereplication, removal of chimeric sequences, grouping into amplicon sequence variants (ASVs) (12), and producing an ASV-per-sample contingency table (12, 13). We used blastn v2.2.31+ to compare ASVs against all NCBI GenBank records as of August 2019 and remove non-fungal sequences. Fungal ASVs were then taxonomically annotated by comparing against the UNITE database of fungal ITS sequences (5) using the Naïve Bayesian Classifier tool (Wang et al. 2007) available in mothur v1.39.5 (7). We removed from the dataset all ASVs found in the negative controls, and then grouped ASVs into operational taxonomic units (OTUs) at a 99% similarity threshold using cd-hit v4.6 (14), with consensus taxonomy assignments per OTU obtained from the ASV annotation with the *classify.otu* tool of mothur. We assessed sampling completeness in individual samples using rarefaction curves built with functions in package vegan v2.5-4 (15) of r v3.6.3 (16). To normalize total read abundances per sample and account for differences in library size, we relied on a mixture model using the variance stabilization method available in the r package DESeq v1.35.1 (17).

**Inoculation of *Arabidopsis thaliana* with fungal isolates.**

We used 2-week-old pure cultures of each isolate growing on corn meal agar (CMA, Sigma-Aldrich, St. Louis, MO, USA) to prepare the fungal inoculum for the *Arabidopsis thaliana* bioassays. Because several isolates did not produce conidia in culture, we relied for inoculation on hyphal rather than on spore suspensions. To do this, we placed in a 2 ml tube four 5-mm-diam. agar plugs taken from the margins of a colony. We washed off possible conidia that could contribute to differences in the amount of inoculum across isolates by washing the plugs three times with 1.5 ml of 0.002% Tween 20 (VWR International GmbH, Darmstadt, Germany), shaking every time for 1 min with a vortex, and discarding the liquid. We then ground the plugs in 1 ml sterilized distilled water with a Retsch MM200 mixer mill (Retsch GmbH, Haan, Germany), and added 50 μl of the suspension to the base of each *A. thaliana* seedling, using a cut pipette tip. In addition, we plated 50 μl from every hyphal suspension on 0.5% (w/v) malt extract agar (MEA, Applichem, Darmstadt, Germany) supplemented with 0.1% (v/v) Triton X-100 (Sigma) and 0.5 gl^-1^ chloramphenicol (AppliChem) to confirm growth of the fungi. To mock-inoculate plants in the negative controls, we followed all the steps described above but using uncolonized CMA plugs.

**Measurement of *Arabidopsis thaliana* total shoot area.**

To measure the total shoot area of *Arabidopsis thaliana* plants growing on clay granules in Magenta™ vessels (18), we took cenital photographs of the open vessels with a Canon PowerShot SX500 IS, always keeping similar conditions of illumination, focal distance, and camera settings. We used the ImageJ software (19) to calibrate the scale of images in millimeters, and to measure total shoot area by detecting green pixels using the tool ‘Color Threshold...’ with the following parameters: Hue, 40–100; Saturation, 0–255; and Brightness, 0–255. Before taking the measurements, we manually erased noise detected around the plants using the ‘Overlay Brush’ tool. We obtained the shoot area per plant by dividing the total area measured by the number of plants in each vessel, which in many cases were less than the three planted due to impaired growth. We tried to differentiate plants overcome by fungal growth (considered in measurements) from those that failed to grow (not considered), based on the presence or lack thereof of shoot stumps in the vessels, respectively. Examples of the detection of greens in images are provided in Fig. S2.

**Total RNA extraction from *Arabidopsis thaliana* shoots.**

We froze shoots of *Arabidopsis thaliana* in liquid nitrogen immediately after collecting them, and then ground them with a Retsch MM200 mixer mill (Retsch GmbH, Haan, Germany), resuspended them in 600 μl TRIzol (Invitrogen, Carlsbad, CA), and stored them overnight at -80 °C. We thawed the samples on ice and added 200 μl of ice-cold chloroform, shook the tubes vigorously, and centrifuged them at 17 000 *g* for 15 min at 4 °C. The nucleic acids in the aqueous supernatant were precipitated with isopropanol at -20 °C for 20–40 min, pelleted by centrifugation at 17 000 *g* for 25 min at 4 °C, washed with 400 μl ice-cold 70% ethanol, and resuspended in nuclease-free water. We digested the DNA in samples using DNase I (New England Biolabs GmbH, Schwalbach, Germany) and then precipitated the RNA using 3 M sodium acetate and absolute ethanol. The pelleted RNA was washed with 70% ethanol and finally resuspended in nuclease free-water. We prepared all reagents used for the extraction with DEPC (Invitrogen)-treated water, and cleaned thoroughly the material and surfaces with RnaseZAP (Sigma-Aldrich, St. Louis, MO, USA) during the process. We measured the quantity and quality of RNA using a Nanodrop spectrophotometer (NanoDrop products, Wilmington, DE, USA), and visualized its integrity by denaturing urea polyacrylamide gel electrophoresis.

**References**

1. Maciá-Vicente JG, Popa F. 2021. Local endemism and ecological generalism in the assembly of root-colonizing fungi. Ecol Monogr 92:e01489.

2. Hopple JS Jr, Vilgalys R. 1994. Phylogenetic relationships among coprinoid taxa and allies based on data from restriction site mapping of nuclear rDNA. Mycologia 86:96–107.

3. de Hoog GS, van den Ende AHGG. 1998. Molecular diagnostics of clinical strains of filamentous Basidiomycetes. Mycoses 41:183–189.

4. Gardes M, Bruns TD. 1993. ITS primers with enhanced specificity for basidiomycetes - application to the identification of mycorrhizae and rusts. Mol Ecol 2:113–118.

5. Kõljalg U, Larsson K-H, Abarenkov K, Nilsson RH, Alexander IJ, Eberhardt U, Erland S, Høiland K, Kjøller R, Larsson E, Pennanen T, Sen R, Taylor AFS, Tedersoo L, Vrålstad T. 2005. UNITE: a database providing web-based methods for the molecular identification of ectomycorrhizal fungi. New Phytol 166:1063–1068.

6. Wang Q, Garrity GM, Tiedje JM, Cole JR. 2007. Naïve bayesian classifier for rapid assignment of rRNA sequences into the new bacterial taxonomy. Appl Environ Microbiol 73:5261–5267.

7. Schloss PD, Westcott SL, Ryabin T, Hall JR, Hartmann M, Hollister EB, Lesniewski RA, Oakley BB, Parks DH, Robinson CJ, Sahl JW, Stres B, Thallinger GG, Horn DJV, Weber CF. 2009. Introducing mothur: open-source, platform-independent, community-supported software for describing and comparing microbial communities. Appl Environ Microbiol 75:7537–7541.

8. Altschul SF, Gish W, Miller W, Myers EW, Lipman DJ. 1990. Basic local alignment search tool. J Mol Biol 215:403–410.

9. Katoh K, Standley DM. 2013. MAFFT multiple sequence alignment software version 7: improvements in performance and usability. Mol Biol Evol 30:772–780.

10. Castresana J. 2000. Selection of conserved blocks from multiple alignments for their use in phylogenetic analysis. Mol Biol Evol 17:540–552.

11. Stamatakis A. 2014. RAxML version 8: a tool for phylogenetic analysis and post-analysis of large phylogenies. Bioinformatics 9:1312–1313.

12. Callahan BJ, McMurdie PJ, Holmes SP. 2017. Exact sequence variants should replace operational taxonomic units in marker-gene data analysis. ISME J 11:2639–2643.

13. Callahan BJ, McMurdie PJ, Rosen MJ, Han AW, Johnson AJA, Holmes SP. 2016. DADA2: High resolution sample inference from Illumina amplicon data. Nat Methods 13:581–583.

14. Fu L, Niu B, Zhu Z, Wu S, Li W. 2012. CD-HIT: accelerated for clustering the next-generation sequencing data. Bioinformatics 28:3150–3152.

15. Oksanen J, Blanchet FG, Friendly M, Kindt R, Legendre P, McGlinn D, Minchin PR, O’Hara RB, Simpson GL, Solymos P, Stevens MHH, Szoecs E, Wagner H. 2019. vegan: Community Ecology Package.

16. R Core Team. 2020. R: A Language and Environment for Statistical Computing. R Foundation for Statistical Computing, Vienna, Austria.

17. Anders S, Huber W. 2010. Differential expression analysis for sequence count data. Genome Biol 11:R106.

18. Kia SH, Pallesch S, Piepenbring M, Maciá-Vicente JG. 2019. Root endophytic fungi show low levels of interspecific competition *in planta*. Fungal Ecol 39:184–191.

19. Abràmoff MD, Magalhães PJ, Ram SJ. 2004. Image processing with ImageJ. Biophotonics 11:36–42.
